# Public attitudes and values in priority setting

**DOI:** 10.1186/s13584-015-0025-8

**Published:** 2015-06-19

**Authors:** Stuart J Peacock

**Affiliations:** Canadian Centre for Applied Research in Cancer Control (ARCC), British Columbia Cancer Research Centre, 675 West 10th Avenue, Vancouver, V5Z 1L3 Canada; Cancer Control Research, British Columbia Cancer Agency, University of British Columbia, British Columbia, Canada; School of Population and Public Health, University of British Columbia, British Colombia, Canada

## Abstract

There is growing recognition that critical decisions concerning investments in new health care technologies and services should incorporate society’s values along with the scientific evidence. From a normative perspective, public engagement can help realize the democratic ideals of legitimacy, transparency, and accountability. On a more pragmatic level, public engagement can help stakeholders understand the degree of popular support for policy options, and may enhance public trust in decision-making processes. To better understand public attitudes and values relating to priority setting in health care, researchers and decision-makers will have to employ a range of quantitative and qualitative approaches, drawing on different disciplines and methodological traditions.

## Public attitudes and values in priority setting

There is growing recognition that critical decisions concerning investments in new health care technologies and services should incorporate society’s values along with the scientific evidence. In many contexts, we are seeing evidence that both decision-makers and the public want the community to be more involved in health care decisions that affect them. Interest in public involvement may be motivated by a number of issues, including public ownership of the health system, concerns over the ability of governments to continue to fund ever increasing levels of service, and legal requirements for public input in decision-making.

In their recent article in the Israel Journal of Health Policy Research, Kaplan and Baron-Epel present findings from a survey on public attitudes on health care priorities at the personal and national level [1]. This survey describes the attitudes (which are sometimes called opinions, preferences, values etc. depending on which discipline the writer comes from) of a representative sample of the Israeli population on different health care priorities from the perspective of the: i) Minister of Health making funding decisions for the whole population; and, ii) the individual choosing what should be included in their personal health insurance package. This represents an interesting addition to the growing literature on public attitudes and values in health, in particular with the paper’s focus on the question: do respondents give different answers from the two different perspectives (which the authors describe as national policy vs. personal needs)? Over half of the respondents (54 %) chose different national priorities from their individual priorities, which the authors conclude meant that over half were able to differentiate between population level policies and their preferences for their own health insurance package. Beyond that, the survey suggests that some of the 46 % of remaining respondents will genuinely believe national policy ‘needs’ coincide with their individual ‘needs’. Therefore, they conclude, the survey shows evidence that the majority of people can understand differences in perspectives and we should continue to look at ways to incorporate public values in health care resource allocation decision-making. That said, the authors argue that surveys and polls may not be the best way forward.

This represents a strong addition to the literature. The authors acknowledge that the survey format did not allow them to fully explore why some respondents answered the way they did, nor will it have fully probed respondents to see if they really understood the nature of the task at hand. But, that is a common weakness of survey approaches, and one which the paper concludes will limit its usefulness in this setting. In what follows, I would like to briefly turn to two hopefully salient issues: i) the normative and pragmatic reasons for eliciting public values relating to priority setting in health care; and ii) deliberative public engagement as a potential way forward.

Eliciting public and patient attitudes and values relating to the adoption of new health care interventions, and publishing information on the evidence that informs funding recommendations is part of a growing international agenda on public engagement and priority setting [2]. At its broadest, we can think of public engagement as including any activity that communicates decisions or involves the public in policy-forming activities [3]. From a normative perspective, there is agreement among many stakeholders that public engagement is necessary to realize the democratic ideals of legitimacy, transparency, and accountability [4, 5]. Furthermore, these ideals have been advanced as necessary for supporting fair and ethical health-care decision-making [6]. On a more pragmatic level, public engagement can help stakeholders understand the degree of popular support for policy options, and may enhance public trust in decision-making processes [2]. However, there is still a lack of practical guidance for integrating values from public engagement with other forms of evidence. Researchers investigating the use of public engagement alongside other evidentiary inputs have shown that public input has been unevenly sought and utilized [7].

Given the growing importance of public engagement, a number of studies have examined the utilization of public input and other types of evidence in health care priority setting [8, 9]. Public engagement methods in health care have traditionally employed one-way communicative or consultative flows of information [7]. One-way communication occurs where an institution provides information about a priority-setting decision. Feedback from the public is not an element of one-way engagement and there is no a priori mechanism to address public concerns should they arise [3]. One-way forms of public engagement include media advertisement and information provided on the internet. A consultative flow of information is where an institution elicits values from the public, but there exists no formal dialogue between the public and stakeholders [3]. Consultative methods for public engagement include focus groups, attitudinal surveys, or opinion polls that elicit values using methods typically developed in economics, psychology or anthropology. These approaches are often ‘one-off’ exercises that stand-alone as measures of ‘acceptability’ or ‘benefit’ to the community.

In recent years, two-way deliberation engagement methods have received significant attention from both researchers and decision-makers [7]. Deliberative public engagement can employ multiple in-person sessions where the public is presented with a variety of perspectives on a given policy topic. Such methods are gaining popularity because they provide an opportunity for the public to express informed judgments. This is pursued through a process that includes relaying traditional scientific evidence in parallel with the public directly communicating their perspectives [11, 12]. Deliberative methods are particularly useful in circumstances where decisions are complex and require informed debate to reach a decision that all parties can agree are reasonable [2]. Equally deliberative methods can be used to emphasize the identification of points of persistent disagreement in participants’ deliberations. This is intended to avoid premature and/or”shallow” consensus and is motivated by an understanding that in some instances differences in underlying values will make it impossible for individuals to agree on certain issues [13].

Deliberative public engagement has significant potential to inform health policy. In particular, the public can play the role of “value consultants” [14] by helping to define policy issues [15] and make value judgments related to the social and normative aspects of scientific or technical issues [16]. Empirical evidence has shown that the public can make coherent and sophisticated recommendations concerning values and health policy and can provide valuable knowledge for decision-makers [17–19]. Public engagement enhances accountability, especially in government decision-making [20] and has been argued to improve the legitimacy of decisions taken [21]. Deliberative methods can also help to build consensus and resolve moral conflict by creating platforms for shared decision-making on key policy issues [22]. In this context, it can be argued that deliberative public engagement methods contribute to decisions that are more likely to be perceived by the public as legitimate, and thus acceptable.

Indeed, Israel has already provided a fertile ground for public engagement in priority setting. In their 2008 paper, Guttman et al. describe the Health Parliament public consultation initiative [23]. This was a large scale public consultation exercise with 132 participants taking place in several regions across the country over several months, yielding a number of recommendations for the Minister of Health and the Health Council. What then is the best way forward for Israel? The answer most likely lies in mixed methods using multidisciplinary approaches. Survey data, such as those provided by Kaplan and Baron-Epel provide much valuable data - especially quantitative data - that can be used to provide valuable insights into public attitudes and values. But, our understanding of what respondents were really thinking when answering questions can be limited. Qualitative methods provide us with approaches than can give us much richer data on respondents attitudes and values, but this in-depth understanding often means we can ask fewer priority setting questions of fewer people. Large scale public consultations are expensive and difficult to undertake in strained economic times. Perhaps what we might be thinking of as a complement to large surveys and consultations are smaller scale deliberative public engagement exercises that probe the public’s attitudes and values on important topics, exercises which take place over a few days with 20–30 participants or so. These methods are being developed elsewhere (O’Doherty & Burgess, 2009; O’Doherty et al. 2012), at least in part to provide a more cost-effective but nonetheless effective approach to deliberative public engagement. Public attitudes and values towards priority setting in health care are diverse and multifaceted; to better understand them researchers and decision-makers will have to use diverse and multifaceted approaches.
